# Isolated Interrupted Aortic Arch: Unexpected Diagnosis in a 63-Year-Old Male

**DOI:** 10.1155/2011/989621

**Published:** 2011-06-21

**Authors:** Hassan Javadzadegan, Jahan Porhomayon, Alireza Sadighi, Mehrdad Yavarikia, Nader Nader

**Affiliations:** ^1^Madani Heart Center, Tabriz Medical University, Tabriz, Iran; ^2^VA Western New York Healthcare System, Division of Critical Care and Pain Medicine, Department of Anesthesiology, State University of New York at Buffalo School of Medicine and Biomedical Sciences, Buffalo, NY 14215, USA; ^3^Tuberculosis and Lung Disease Research Center, Tabriz Medical University, Tabriz, Iran; ^4^Cardiology Division, Tabriz Medical University, Tabriz, Iran; ^5^Surgery and Pathology VA Western New York Healthcare System, Division of Cardiothoracic Anesthesia and Pain Medicine, Department of Anesthesiology, State University of New York at Buffalo School of Medicine and Biomedical Sciences, Buffalo, NY 14215, USA

## Abstract

A 63-year-old male with history of hypertension, dyspnea on exertion, and chronic chest pain was admitted for elective cardiac angiography. Arterial blood pressure was 160/90 mmHg in both arms. Femoral and popliteal pulses were extremely weak, and third (S_3_) and fourth (S_4_) heart sounds were audible. Aortography showed a mildly dilated aortic root with double brachiocephalic trunk and interruption of aortic arch at isthmus. Profuse and well-developed collaterals appeared at neck and thorax. The patient was recommended to take medical treatment for his hypertension and advanced heart failure. The aim of this paper, is to review the diagnostic and therapeutic options for treatment of the interrupted aortic arch.

## 1. Introduction

Interrupted aortic arch (IAA) is a very rare congenital malformation associated with other congenital cardiac defects. It is defined as complete absence of luminal continuity between the ascending and descending portions of the aorta [1–4]. IAA is rare in adult patients. The majority of patients die from this anomaly before adulthood [2–6].

## 2. Case Report

In April 2009, a 63-year-old man presented to our outpatient clinic with the chief complaint of fatigue, dyspnea, and chest pain for a period of six months with regular activity. He became increasingly symptomatic one month prior to his visit to the clinic. He had history of hypertension and progressive dyspnea for four years evident from his medical records. Social history was significant for smoking. On exam, his arterial blood pressure was 160/90 mmHg on upper extremity and extremely weak lower extremities femoral and popliteal pulses. Dorsal is pedis and tibialis anterior pulses were not palpable. Two-plus pitting edema was present in lower extremities. S_3_ and S_4_ were audible. Breath sounds were clear on auscultation. chest radiography showed increase cardiothoracic ratio. 

**Figure 1:**
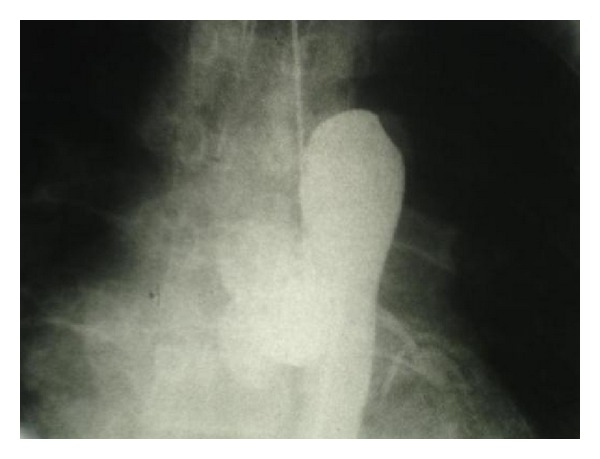
Complete occlusion of proximal portion of descending aorta.

**Figure 2:**
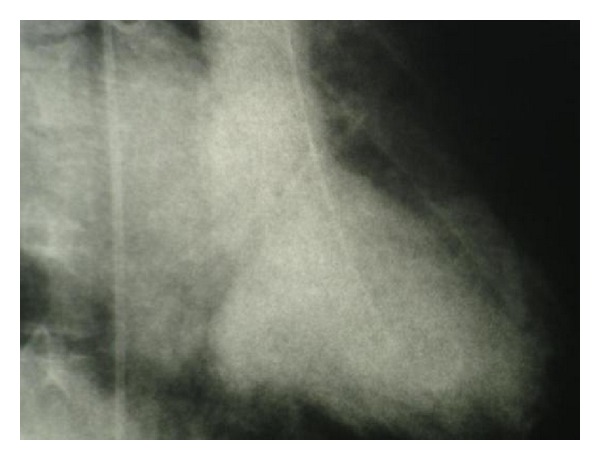
LV angiogram.

**Figure 3:**
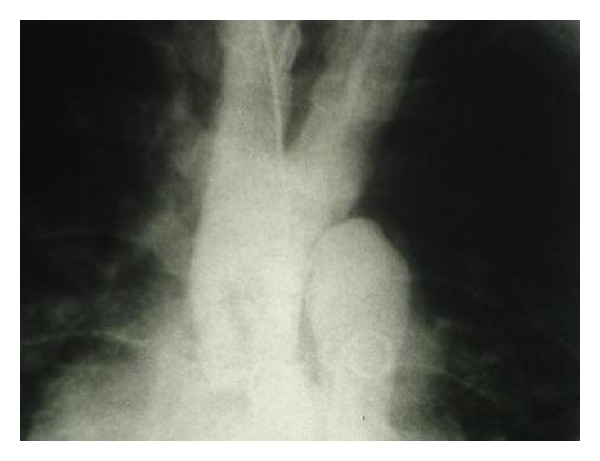
Double brachiocephalic trunk.

**Figure 4:**
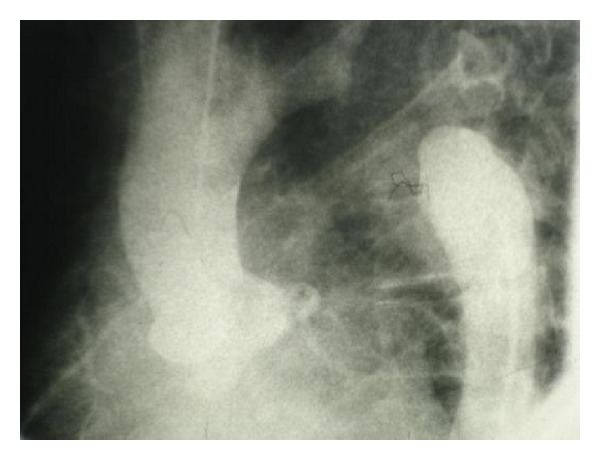
Complete interruption of Aorta.

**Figure 5:**
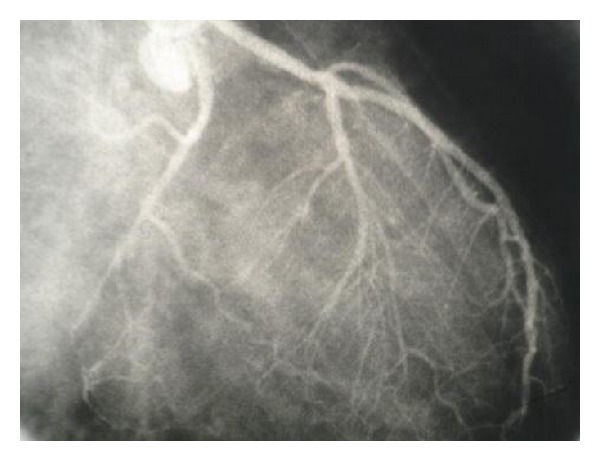
Left coronary artery with minimal narrowing.

**Figure 6:**
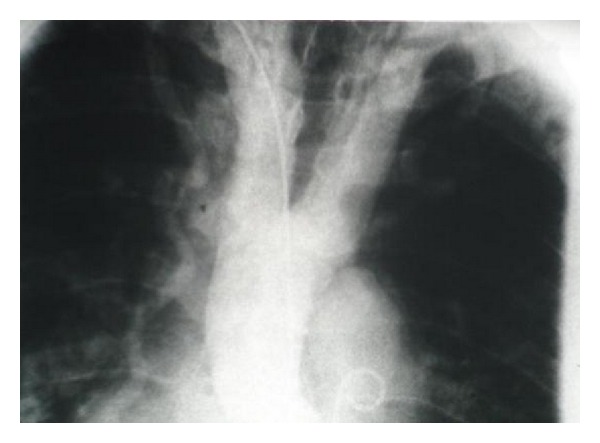
Well-developed collaterals (dilated right internal mammary artery).

The patient was scheduled for cardiac catheterization. During cardiac angiography, we attempted to pass a guidewire to the aortic arch from right femoral artery without success. Radio contrast injection revealed complete occlusion of proximal portion of descending aorta (Figure 1). We then proceeded with catheterization via right brachial artery, left ventricular dye injection, and selective coronary angiography. The study revealed moderately enlarged left ventricle (LV) cavity with severely depressed LV contraction with global hypokinesia, (Figure 2). Estimated Left Ventricular Ejection Fraction (LVEF) was about 20%. Aortography revealed mildly dilated ascending aorta with double brachiocephalic artery trunk and interrupted aortic arch in the isthmus (Figures 3 and 4). Evaluation of coronary vessels showed no obstructive lesion (Figure 5). Profuse and well-developed collateral vessels were observed in the lower neck and upper thoracic region (Figure 6).

Patient was referred to cardiothoracic surgery service for surgical correction. Patient refused surgery. He was subsequently followed with the cardiology service for the next Ten months. He expired at home from myocardial infarction.

## 3. Discussion

IAA is a very rare congenital anomaly that occurs in 3 per million live births and accounts for 1% of all congenital heart diseases [2, 7]. Clinical presentation of IAA is discovered at infancy. IAA is associated with severe congestive heart failure. A ventricular septal defect is usually present as well as a patent ductus arteriosus. Patent ductus arteriosus is needed for perfusion of the descending aorta. The development of collateral vessels is progressive. Death within the first days of life is due to closure of the patent ductus arteriosus. Spontaneous closure of a muscular ventricular septal defect is always possible as well as a progressive stenosis of the ductus arteriosus. If left ventricle is untreated, 90% of the affected infants die at a median age of 4 days [4, 8]. 

Based on Celoria and Patton classification, IAA is divided into 3 types:

1. discontinuity distal to the left subclavian artery (type A, 43% of cases);
2. interruption between the left carotid and left subclavian arteries (type B, 53% of cases);
3. interruption between the innominate and left carotid arteries (type C, <4% of cases) [9].

Genetic predisposition has been suggested for the etiology of IAA type B [10–12].

DiGeorge syndrome has been found in the majority of IAA type B patients and screening for this syndrome is recommended [6, 10].

Careful physical examination of the lower- and upper-peripheral pulses in young adults with chief complain of hypertension is of outmost importance. It provides the first clue to the diagnosis of IAA and coarctation of aorta. Currently, noninvasive diagnostic techniques such as echocardiography, computed tomography, and magnetic resonance imaging of heart are preferred tools for diagnosis of IAA [13]. Our patient, presented with history of chest pain and catheter angiography, was the first preferred diagnostic modality and IAA was an incidental finding. We suspected coarctation of aorta because of the difference between upper and lower extremities arterial blood pressure and pulses. Cardiac catheterization supported the diagnosis of IAA. In addition, profuse collaterals along with loss of antegrade flow to descending aorta and bifid brachiocephalic artery were in favor of IAA. Diagnosis IAA and coarctation of aorta are suggested with the presence of filling defect in the descending aorta and failure of contrast to communicate with the proximal portion of aortic arch. Surgical correction of this anomaly is the definite treatment [1–3, 8]. Incidental diagnosis of IAA in persons older than 50 years old associated with cardiac symptoms raises an important question on benefit of surgery. Patients diagnosed with interruption of the aortic arch in adulthood might be displaying progression of undiagnosed coarctation of aorta. Three-dimensional computed tomography is useful to detect the obstructive lesion and to guide the surgical approach [12].
